# 

**Published:** 1842-06

**Authors:** 


### WESTERN JOURNAL

OF

### MEDICINE AND SURGERY.

### EDITORS.

---

DANIEL DRAKE, M. D.,

PROFESSOR OF PATHOLOGICAL ANATOMY AND CLINICAL MEDICINE IN THE MEDICAL INSTITUTE OF LOUISVILLE:

LUNSFORD P. YANDELL, M. D.,

PROFESSOR OF CHEMISTRY AND PHARMACY IN THE SAME:

THOMAS W. COLESCOTT, M. D.

### INDEX TO VOL. V.

A

Absence of the uterus, 198.
Abundance of food and mortality, connection between, 233.
Action of spirits upon habitual drunkards, 195.
Acupuncture in reduction of strangulated hernia, 225.
Alleged discharge of hairs from the hand, 80.
Anchylosis of the lower jaw with the temporal bones, 367.
Antidote to the salts of copper, 221.
Arsenic and the trembles, 236.
Asphyxia from drowning, treatment of, 230.
Asphyxia of infants, treated by cold dash, 398.
Asthma, use of quinine in, 382.
Asthma, thymic, case of, 427
Atmosphere, constitution of, 227.
Auscultation and percussion, rectifications in the practice of, 383.

B

Barton's operation for cure of deformed leg, 149.
Bell's Report of the McLean Asylum for the insane, 271.
Belladonna in paraphimosis, 77.
Bibliographical notices and reviews, 129, 139, 141, 267, 271, 278, 282, 355, 357, 429.
Bilious remittent fever, in Wabash Valley, &c., 42.
Blind, Kentucky School for instruction of, 237, 317.
Blind, Ohio School for instruction of, 240
Blindness, division of the muscles of the eye in certain cases of, 302.
Blythe, Dr. James, death of, 474.
Botany, new works on, 400.
Burns, treatment of, 74.

C

Calomel reduced to the protoxide in the primæ viæ, 159.
Canula of lead in fistula in ano, 399.
Carroll on the topography, &c., of a district in Ohio, 20.
Casein, albumen, &c., as elements of nutrition, 294.
Cesarean operation terminating fatally, 352.
Children at the breast, pulse of, 294.
Cicuta in gonorrhœa, 76.
Clavicle, dislocation of backwards, 314.
Club-foot, operation for, 395.
Cold Dash in the asphyxia of infants, 398.
Composition of false membranes, 200.
Conception, physiology of, 204.
Conditions which favor an excessive size of the fœtus, 226.
Condition of the nails in fractured limbs, 77.
Conium and opium, 73.
Connection between abundance of food and mortality, 233.
Constitution of the atmosphere, researches into, 227.
Convention, Medical, of Ohio, 317, 469.
Copper, antidote to salts of, 221.
" detection of, 223.
Copy-right in lectures, 233.
Correction, 400, 476.
Cure for the bite of a snake, 394.
Creosote, in hemorrhage after lithotomy, 225.
Crystalline lens, dislocation of, 307.
Cystitis, chronic, case of, 41.

D

Dawson's notes on scarlet fever in Green County, Ohio, 126.
Death from erysipelas, 391.
" of Dr. Blythe, 473.
Deformed leg from fracture, operation for cure of, 149.
Dennis' case of thymic asthma, 427.
Detection of arsenic acid, 293.
" of copper, new process for, 223.
Diagnosis of inflammation of the grey and white matter of the brain, 206.
Diarrhœa, treated by monesia, 297.
" and dysentery, use of nitrate of silver and ipecacuanha in, 383.
Digitalis, 157.
Disease of the tongue and mouth, 319.
Dislocation of the crystalline lens, 307.
" of the clavicle backwards, 314.
Division of the muscles of the eye in certain cases of blindness, 302.

Dropsy, use of quinine in, 76.
Drowning, treatment of asphyxia from, 230.
Drunkards, action of spirits upon, 195.
Dry gangrene, 397.
Dubois on the auscultatory signs of pregnancy, 301.
Dunglison's Practice of Medicine, 282, 429.
Dysentery, 383, 398.

E

Editorial department, 79.
" notice, 317.
Elements of surgery, by Liston, new edition of, 399.
Erysipelas, death from, 391.
Ergot, 212.
Espy's Philosophy of Storms, 129.
Evolution of tubercle, 201.
Explanation, 157.
Extraction of foreign bodies from the ear, 368.

F

Facts and circumstances relating to a case of compound fracture, and suit for malpractice, 141.
False membranes, composition of, 200.
Fatal hemorrhage from extraction of a tooth, 377.
Fever, (bilious remittent,) as it occurs in the Wabash valley, 42.
Fever, (yellow,) of Natchez and of the South-West, 161, 321, 401.
Feigned hysteria, 213.
Ferguson's Hospital Reports, 1.
Fibre, 372.
Fibrin, albumen, casein, as elements of nutrition, 294.
Fistula in ano, leaden canula in the treatment of, 399.
Fœtus, conditions favoring an excessive size of, 226.
Food and mortality, connection between, 233.
Foreign bodies removed from the ear by syringing with cold water, 368.
Foreign bodies in the air passage for nine months—expulsion, 457.
Fractured bones, union of, 211.
Fracture of the neck of the femur within the capsule, followed by osseous union, 457.

G

Gonorrhœa, extract of circuta in, 76.
Gross' Lecture on Strabismus, 241.
Guardian of Health, 139.

H

Hall (Marshall) on the use of setons, 207.
Hardin on phlegmasia dolens, 265.
Harper's case of chronic cystitis, 41.
Head, injury of, with loss of portion of the brain, 39.
Health, injuries to from breathing impure air, 310.
Heart, sounds of, 383.
Hemorrhage from extraction of a tooth, 377.
Hemorrhage after lithotomy, 225.
Hernia, new variety of, 369.
" case of inflamed, 458.
" simple inflammation of, 460.
Holme's case of uterine hemorrhage, 192.
Human placenta, structure of, 379.
Humbug and quackery, 292.
Hydrophobia, idiopathic, 467.
Hysteria, feigned, 213.
" use of tobacco in, 465.

I

Idiopathic hydrophobia, 467.
Impropriety of mercurial salivation, 183.
Infants, cold dash in the asphyxia of, 398.
Influence of the last inundation on the health and population of Lyons, 228.
Inflammation, 292.
" of the grey and white matter of the brain, 206.
Inflammation of hernia, 460.
Injury of the head with loss of portion of brain and recovery, 39.
Injuries to health occasioned by breathing impure air in close apartments, 310.
Insane, what should be done with them, 81.
Ioduret of sulphur in porrigo, 388.

J

Jarvis on the treatment of the insane, 81.

K

Kentucky school for the instruction of the blind, 237, 317.
Kentucky Lunatic Asylum, 238.
" State medical society, 79.
Kiestein as an evidence of pregnancy, 299.

L

Labor, case of, attended by some unusual appearances, 15.
Lapis divinus in otorrhœa, 305.
Leaden canula in the treatment of fistula in ano, 399.
Lectures, copyright in, 233.
Liston's elements of surgery, new edition of, 399.
Lithotomy, 80, 159.
" at Naples, statistics of, 224.
Lugol's lectures on scrofula, 285.
Lunatic asylum of Kentucky, 238.
Luxations of the thigh, reduction of, 210.

Lyons, influence of the last inundation on the health and population of, 228.

M

Malformation of the shoulders, 451.
Malgaigne on pseudo-strangulation, or simple inflammation of hernia, 460.
Malpractice, report of the facts and circumstances relating to a prosecution for, 141.
Measles, French, 397.
Medical convention of Ohio, 317, 469.
" " " " proceedings of, 357.
Medical quarrels, 226.
Medical schools of the west, 473.
Medusæ, stinging organs of, 463.
Membranes, false, their composition, 200.
Memoir of a gentleman born blind and successfully operated on in his 18th year, 308.
Menstruation and uterine hemorrhage, singular phenomena accompanying, 69.
Menstruation, physiology of, 203.
Mercurial salivation, impropriety of, 183.
Mesenteric glandular affections, treatment of, 452.
Milk, researches on, 296.
Milk-sickness and the trembles, 235.
Misrepresentations, 317.
Monette on the epidemic yellow fever of Natchez, 161, 321, 401.
Mortality and abundance of food, connection between, 233.
Mouth, disease of, 319.

N

Nails, condition of in fractured limbs, 77.
Natchez, epidemic yellow fever of, 161, 321, 401.
Naples, statistics of the lateral operation of lithotomy at, 224.
Needle and twisted suture in the treatment of varicose veins, 288.
Neuralgia, beneficial effects of narcotism in some obstinate cases of, 215.
New medical journal in Boston, 297.
New process for the detection of copper, 223.
New variety of hernia, 369.
New works on botany, 400.
Nitrate of silver and ipecacuanha in diarrhœa, &c., 383.
Notes on the scarlet fever, as it appeared in Green county, Ohio, 126.

O

Observations on the topography, climate and diseases of a district in the eastern part of Ohio, 20.
Observations on the epidemic yellow fever of Natchez, 161, 321, 401.
Obstetric medicine and surgery, principles and practice of, 278.
Ohio lunatic asylum, 237.
" medical convention, 317, 469.
" school for the blind, 240.
Operation for club-foot, 395.
" for cure of deformed leg, 149.
Opium and conium, effects of, 73.
Opium in acute internal inflammations, 216.
Otorrhœa, lapis divinus in, 305.
Overton's case of labor, 15.
Ozœna, cure of, 369.

P

Paraphimosis, extract of belladonna in, 77.
Past winter and present spring, 393.
Periodicals, western, 472.
Pharmacy in France, 389.
Philosophy of storms, 129.
Phlegmasia dolens, 265.
Physiological temperance society, 80.
" " " proceedings of, 267.
Physiology of menstruation, 203.
" of conception, 204.
Poisoning from the use of tobacco enema, 72.
Polypus uteri, 159.
Porrigo, ioduret of sulphur in, 388.
Pregnancy, Kiestein as an evidence of,
" auscultatory signs of, 301.
Prevention of tubercles, 216.
Principles and practice of obstetric medicine and surgery, 278,
Proceedings of the medical convention of Ohio, 357.
Process for the detection of copper, 223.
Progress of total abstinence, 394.
Prolapsus uteri, of sixteen years standing, reduced, 211.
Prosecution for malpractice, 141.
Pseudo-strangulation, or simple inflammation of hernia, 460.
Pulse of children at the breast, 294.

Q

Quackery and humbug, 292.
Quarrels, medical, 226.
Quinine in dropsy, 76.
" in asthma, 382.

R

Raciborski on the physiology of menstruation, 203.
Ramsbotham's principles and practice of obstetric medicine and surgery, 278.

Rectifications in the practice of auscultation and percussion, 383.
Reduction of a strangulated hernia, 225.
" of luxations of the thigh, 210.
" of a prolapsus uteri after sixteen years continuance, 211.
Regeneration and union of nerves, 370.
Relapse in intermittent fever, remedies against, 215.
Remarks on a review of an essay on trembles, &c., 64.
Report on the treatment of burns, 74.
" of the facts and circumstances relating to a case of compound fracture and prosecution for malpractice, 141.
Report of the McLean asylum for the insane, 271.
Report on the establishment of an asylum for the insane in Indiana, 355.
Report of the Louisville marine hospital, 1.
Reviews and bibliographical notices, 129, 139, 141, 267, 271, 278, 282, 355, 357, 429.
Researches into the real constitution of the atmosphere, 297.
Researches on the function of the skin in man and animals, 371.
Rheumatism of the skin, 71.
Rotatory movements of the yolk in the ovum of mammalia, during its passage through the fallopian tube, 199.

S

Salts of copper, antidote to, 221.
Sanicula Marilandica, a cure for snake-bite, 394.
Scarlet fever, notes on, 126.
School for the blind in Louisville—petty misrepresentations, 317.
Scrofulous affections, treated by preparations of walnut leaves, 215.
Scrofula, Lugol's lectures on, 285.
Shipman's report of the facts and circumstances, &c., 141.
Shoulders, malformation of, 451.
Setons, clinical remarks on the use of, by Dr. Marshall Hall, 207.
Singular phenomena accompanying menstruation and uterine hemorrhage, 69.
Smick on bilious fever, 42.
South-West, epidemic yellow fever of, 161, 321, 401.
Sounds of the heart, 383.
Spirits, action of upon habitual drunkards, 195.
Statistics of the lateral operation of lithotomy at Naples, 224.
State of the urine during pregnancy and disease, 304.
Stinging organs of the medusæ, 463.
Storms, Espy's Philosophy of, 129.
Strabismus, Gross' Clinical Lecture on, 241.
Strangulated hernia, reduction of apparently effected by acupuncture, 225.
Structure of the human placenta, 379.
Supposed polypus nasi, 381.
Surgery, new edition of Liston's elements of, 399.

T

Thymic asthma, 427.
Tobacco enema, poisoning from the use of, 72.
Tobacco in hysteria, 465.
Todd's case of injury of the head, 39.
Tongue and mouth, disease of the, 319.
Tooth, fatal hemorrhage following extraction of, 377.
Townsend on the impropriety of mercurial salivation, 183.
Total abstinence, progress of, 352.
Trembles and milk-sickness, 235.
" and arsenic, 236.
Tubercle, evolution of, 201.
" prevention of, 216.
Tubular pregnancy, recovery from by the artificial production of abortion, 464.
Twenty-fourth Report of the McLean Asylum for the Insane, 271, 271.

U

Union of fractured bones, 211.
" of nerves, 370.
Urine, state of during pregnancy and disease, 304.
Uterus, absence of, 198.
Uterine hemmorhage, with expulsion of ovum, 192.
Uterine hemorrhage, singular phenomena accompanying, 69.

V

Varicose veins, treatment of by needle and twisted suture, 288.
Vapors of antimony, poisoning by, 214.

W

Walnut leaves, preparations of in scrofulous affections, 215.
Western periodicals, 472.
Winter, the past, 393.
Works on botany, 400.

Y

Yellow fever of Natchez and of the south-west, 161, 321, 401.
Yolk, rotatory movements of in ovum of mammalia during its passage through the fallopian tube, 199.

### RECEIPTS FOR THE MEDICAL JOURNAL,

FOR MAY, 1842.

| | | |
|---|---|---|
| Dr. | S. Loving, Paris, Tenn., - - - | $5 00 |
| | E. Foster, Louisville, Miss., - - - | 5 00 |
| | B. L. Hatch, Aberdeen, Miss., - - - | 10 00 |
| | W. W. Sentney, Falmouth, Ky., - - | 13 00 |
| | C. J. Clark, Jacksonville, Ala., (discount 75 cents,) | 5 00 |
| | Nuckoll, Wadesboro', Ky., - - - | 5 00 |
| | C. A. Trimble, Chillicothe, O., - - - | 5 00 |
| | H. Hard, Plymouth, Ia., - - - - | 5 00 |
| | H. B. Flippen, Celina, Tenn., - - - | 3 00 |
| | P. Capshaw, Whitesburgh, Ala., (dis. 75 cents,) - | 4 25 |
| | H. Howey, New Boston, Ill., - - - | 5 00 |
| | W. D. Ellis, Hernando, Miss., - - - | 5 00 |
| | N. C. Sherrill, Carrollton, Ky., - - - | 5 00 |
| | R. Steele, Paris, Ill., - - - - | 5 00 |
| | A. B. Shipman, Cortland Village, N. Y., - | 5 00 |
| | G. Follis, Pleasureville, Ky., - - - | 5 00 |
| | S. Haines, Owensboro,' do - - - | 5 00 |
| | D. E. Gibson, Bellmont, Tenn., - - - | 5 00 |
| | A. C. Lewis, Scott P. O., Ohio, - - - | 5 00 |

### DELINQUENT LIST.

Below we commence a list of those subscribers, who having taken this Journal from its commencement, have not yet made a single payment. The main design of publishing this list is, to bring it forcibly to the minds of subscribers that they have not paid. Upon this hint, of course every one of them that has the least regard for his honor, will remit the amount he owes, under the post-office frank. In our next, we shall complete the list of this class of delinquents. In the succeeding number, we shall publish the whole of such as do not speak on this hint; and shall keep the list standing, striking off from time to time such as pay up. In two or three months the honest will be pretty effectually winnowed from the dishonest, and those who remain on the list will have a diploma which will distinguish them far beyond any they may have received from learned institutions.

In two or three months we shall commence a list of all those who may fail to remit after their accounts have been presented through the post office. In due time the public will be furnished with a very ready means of ascertaining the character of western physicians.

TENNESSEE.

J. Drake, South Carroll.
J. J. Gibson, Hampshire.
Wilson & Bearden, Petersburgh.
L. M. N. Cook, Silver Spring.
T. J. Walton, Mulloy's.
S. B. Robinson, Versailles.
J. M. Dean, Lynchburgh.
A. F. Clement, Lexington.
W. H. Warren, do
W. T. Eatherty, Rural Hill.
J. A. Lecky, Ripley.
S. Richardson, Rutherford.
W. D. Lee, do.
W. J. J. Morrow, Chattawogga.
J. W. Koen, Colliersville.
J. M. M. Cornelius, Germantown.
Robinson & Hagan, Carthage.
W. D. Dorris, Nashville.
J. B. Copeland, Nolandsville.
R. Forbis, Elkton.
M. W. O'Brien, Springfield.
W. J. C. Rogers, Murfreesboro'.

NEW YORK.

Dr. Wm. Welch, Penfield.
Dr. G. Standing, Utica.

ARKANSAS.

W. A. Threlkeld, Helena.
J. H. McCarty, Ozark.
Dr. Beach, Lawrenceville.
J. Eaton, Pine Bluff.

LOUISIANA.

B. B. Smith, Shreveport.
J. B. Henderson, New Orleans.
J. Davis, Millekin's Bend.
T. Ingersoll, Cherryville.
E. C. Kittredge, Assumption.
E. Ragan, Columbia.

ALABAMA.

W. E. Murphy, Somerville.
P. L. Gilbert, Isny, Washington Co.
R. L. Graves, Leesville.
D. W. Ford, Meridianville.
E. M. Hussey, Waterloo.
Dr. J. D. Sale, Huntsville.
W. R. Davie, do
W. F. Johnson, Benton.
J. Durno, Mobile, or Alton, Ill.
J. C. Harris, Cedar Bluff.
J. T. Reese, Syllacogga.

MISSISSIPPI.

J. B. Scott, Oakland.
Harris & McLaen, Wahallock.
S. Cook, Hernando.
E. F. Bouchelle, Columbus.
S. D. Kennedy, Holmesville.
D. B. Nailer, Vicksburgh.
—— Hogan, Panola.
A. P. Reed, Fort Adams.
J. A. Hodges, Garlandsville.
O. S. Echols, Hamilton.
L. Shelby, Warrenton.

ILLINOIS.

O. Abbey, Quincy.
W. H. Efner, Lacon.
J. W. Chenoweth, Auburn.
C. L. Duncan, Marshall.
J. R. Colwell, Farmington.
J. Kirkpatrick, Fox P. O.
W. Allison, Paradise.
W. Patton, Oakland.
—— Adams, Prarieton.
T. J. Beckwell, Rushville.
D. S. Smith, Juliet.
J. Jayne, Carlinsville.
B. F. Edwards, Alton.
C. F. Falley, York.
H. Symmes, Equality.
W. W. Higgins, Jones' P. O.
W. O. Chamberlain, Princeton.
Wood & Goodwin, Holland's Grove.
F. Canterbury, Cole's C. H.
H. P. Griswold, Plymouth.
J. M. Metcalf, Winchester.
W. Kellar, Decatur.

